# Effect of oral alpha-lipoic acid (ALA) on the treatment of male infertility: A protocol for systematic review and meta-analysis

**DOI:** 10.1097/MD.0000000000018453

**Published:** 2019-12-20

**Authors:** Liang Dong, Xiaojin Zhang, Fang Yang, Junjun Li, Xujun Yu, Yulin Li

**Affiliations:** a Department of Andrology, The Reproductive & Women-Children Hospital, Chengdu University of Traditional Chinese Medicine,; b Chengdu University of Traditional Chinese Medicine,; c Hospital of Chengdu University of Traditional Chinese Medicine, Chengdu, Sichuan, PR China.

**Keywords:** alpha-lipoic acid (ALA), male infertility, meta-analysis, protocol, sperm, systematic review

## Abstract

**Background:**

Male fertility has gradually become a worldwide problem. Because of the limitation of treatment, many drugs have been used for improving sperm quality. Among them, alpha-lipoic acid (ALA), as a treatment of diabetic neuropathy, has been applied to improve the quality of sperm in clinical practice, with satisfactory effect. However, there is still no systematic review on the field of male infertility treating with oral ALA.

**Methods:**

The databases of MEDLINE, EMBASE, Web of Science, Clinicaltrials.org., China National Knowledge Infrastructure Database (CNKI), China Biology Medicine Database (CBM), Wan fang Database, VIP Science Technology Periodical Database, and Cochrane Library were retrieved. Grey literature will be searched in OpenGrey. Related Randomized controlled trials (RCTs) will be collected and selected before December 30, 2019. We will search English literature and Chinese literature using search terms including “male infertility”, “semen”, “sperm”, “alpha-lipoid acid”, “ alpha lipoid acid”, “lipoid acid”. We will start to search database in November 20, 2019. Sperm concentration, motility and morphology, sperm DNA fragmentation index, sperm number of per ejaculate, sperm viability and adverse events will be evaluated. RevMan 5.3 and Stata 14.0 will be used for Systematic review and Meta-analysis. This protocol reported in accordance with the Preferred Reporting Items for Systematic Reviews and Meta-Analyses Protocols (PRISMA-P) statement, and we will report the systematic review by following the PRISMA statement.

**Results:**

Through systematic review, and meta-analysis when necessary, we can obtain the effect of ALA on sperm quality, including sperm motility, concentration, morphology and other indicators.

**Conclusion and dissemination:**

Efficacy and safety of oral ALA on male sperm quality in infertile men will be assessed. The results will be published in a public issue journal to provide evidence-based medical evidence for urologists and andrologists to make better clinical decisions.

## 1 Introduction

More than 15% of married couples suffer from various fertility problems in the world, resulting in infertility. About 50% are caused by men.^[1]^ In China, the sperm quality of men is decreasing by 1% every year.^[2]^ Because there is no specific drug, antioxidants are currently one of the commonly used drugs for the treatment of male infertility.^[3]^ Alpha-lipoic acid (ALA), which can prevent metabolic and genital morphological changes in diabetic individuals,^[4]^ is currently the most potent fat- and water-soluble antioxidant drug, maintains sperm motility and viability by reduction in reactive oxygen species (ROS) production and can also protect sperm DNA integrity.^[5]^ It was attributed to free radical activities. A research has shown that ALA could improve the sperm motility rate and reduce sperm DNA damage, thereby improve sperm quality.^[6]^ Since there is no systematic review about ALA treating male infertility, so we begin to do this work.

## 2 Review objectives

The purpose of this study is to evaluate the effect of oral ALA on male infertility, including sperm concentration, motility and morphology, sperm DNA fragmentation index, sperm number of per ejaculate, etc. The results will provide better clinical decisions for andrologists and urologists.

## 3 Methods

This is a systematic review, with meta-analysis if necessary. The data and results used in this paper are almost from published studies, and there is no ethical issue, so the approval of the ethics committee is not required.

### 3.1 Protocol and registration

This study is registered on PROSPERO. Registration number: PROSPERO CRD42019145592.

This protocol refers to the statement of Preferred Reporting Items for Systematic Reviews and Meta-Analyses Protocols (PRISMA-P).^[7,8]^ And we will report the systematic review in accordance with the PRISMA statement strictly.

### 3.2 Data source

#### 3.2.1 Electronic search database and approach

The electronic databases of MEDLINE, Web of Science, EMBASE, Clinicaltrials.org., China Biology Medicine Database (CBM), China National Knowledge Infrastructure Database (CNKI), Wan fang Database, VIP Science Technology Periodical Database, Chinese Clinical Trial Registry and Cochrane Library were retrieved. Grey literature will be searched in Open Grey. Related Randomized controlled trials (RCTs) will be collected and selected before December 30, 2019. The searching work will be started in November 20, 2019, and updated before the final work completed.

We will choose Medical Subject Heading or text key words “male infertility” or “sperm” or “semen” AND “oral alpha-lipoic acid” or “alpha-lipoid acid” or “alpha lipoic acid” or “lipoid acid”, and different search strategies to fit different databases. Chinese form of the above terms will be used in Chinese search. A specific search example for MEDLINE is shown in Table 1.

**Table 1:**
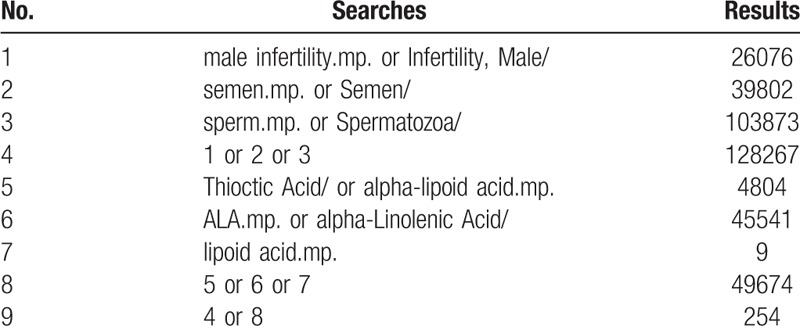
MEDLINE search strategies.

#### 3.2.2 Other sources of search

Grey literature will be retrieved through Open Grey. Full texts will be obtained through library interlibrary loan or purchase. Manual review of references in published articles will be conducted to identify other relevant studies.

### 3.3 Included and excluded criteria

#### 3.3.1 Study design

Only RCTs will be included in this study. Observational studies, retrospective analyses, self-controlled trials, patient series, case reports, reviews, animal studies, laboratorial and in vitro studies, will be excluded.

#### 3.3.2 Participants

*Included population.* Men of childbearing age who have fertility requirements and semen samples at baseline met the World Health Organization (WHO) 1999 criteria (sperm concentration of 20 million/ml (geometric mean of two samples), 50% motile sperm, 15% sperm with normal morphology, and semen volume 1.5 ml (arithmetic mean of two samples))^[9]^ and 2009 criteria (sperm concentration of 15 million/ml (geometric mean of 2 samples), 32% PR sperm, 4% sperm with normal morphology, and semen volume 1.5 ml (arithmetic mean of 2 samples)).^[10]^

*Excluded population.* Undiagnosed patients; female infertility patients; azoospermia; infertility result from hypothalamic-pituitary-gonadal lesion, chromosomal or genetic lesion, hormone abnormality, congenital diseases, obstructive diseases.

#### 3.3.3 Interventions

Randomized, double-blind, placebo-controlled trials would be identified the best.

Treatment group: This group was treated with oral ALA. The patients may have diabetes mellitus, but should not affect vaginal insertion. In addition to abnormal sperm parameters, other causes of male infertility were excluded.

Control group: a placebo with the same appearance as the treatment group.

#### 3.3.4 Outcomes

Primary outcome indicator.

(1) Sperm motility: spermatozoa with activity of A and B levels or spermatozoa with forward-moving sperm in the WHO classification will be included, which provided as a percentage (%).
(2) Sperm concentration: number of sperm per milliliter (× 10^6^/mL).^[11]^
(3) Sperm morphology: proper sperm ratio, provided as a percentage (%).^[11]^
(4) It will be based on the results reported at the end of included studies.

#### 3.3.5 Secondary outcome indicators:

(1) Sperm DNA fragmentation index: Sperm DNA damage was reported in the study. The detection method may be sperm chromatin structure assay (SCSA), terminal deoxyuridine nick end labelling (TUNEL) assay, Comet assay, sperm Chromatin Dispersion (SCD) assay, Acridine orange (AO) test, Aniline blue (AB) staining, Toluidine blue, Chromomycin A3 (CMA3) staining.^[12]^
(2) Sperm number per ejaculate: The total number of sperm contained in once ejaculation (× 10^6^/once ejaculation).
(3) Sperm viability: Proportion of all active sperm (including A, B, C or PR, NP), provided as a percentage (%).
(4) Adverse events: all adverse events, including nausea, vomiting, facial flushing, increased heart rate and other adverse events.

### 3.4 Selection of studies and data extraction

Document management will be conducted by Endnote X9 software. The software will be used to filter duplicate studies first, and then delete duplicate researches by reading titles, abstracts and other relevant information.

According to the inclusion criteria and exclusion criteria, the literature will be further screened. In this process, the controversial literature will be screened after obtaining the full text. Further detailed screening and data extraction of the researches will be carried out simultaneously by two professionally trained reviewers (Junjun Li, Liang Dong).

**Figure 1:**
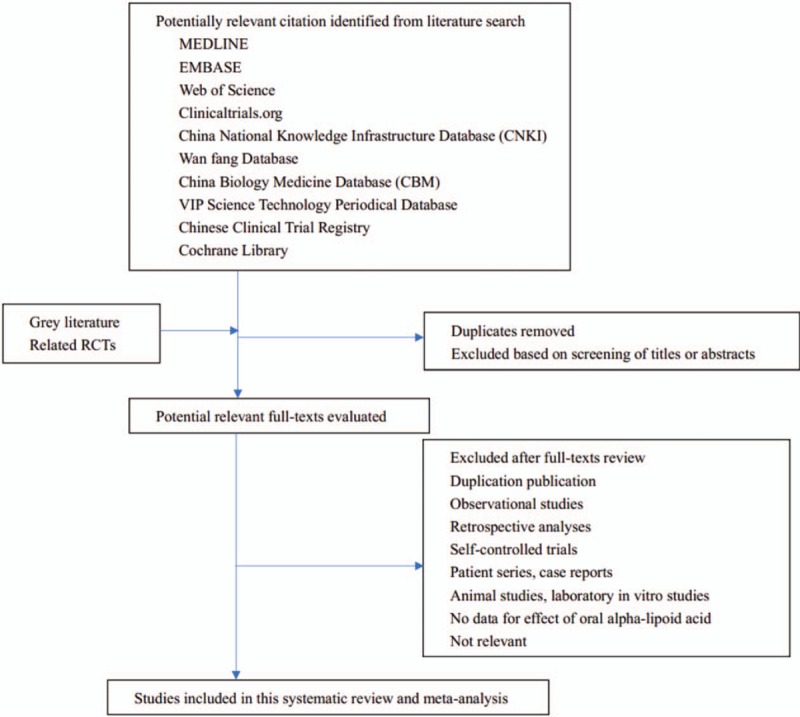
The PRISMA literature screening flow chart.

Then, the studies that meet the inclusion criteria are full-text read and re-screened. If two or more articles have repeated or staged research results, only these articles with the largest sample size, the most complete intervention and longest follow-up time are included. When the review team cannot confirm the repeated studies, the study author will be contacted for judgment. The flow chart of literature screening is shown in Figure 1. All randomized controlled trials which compares oral AL with placebo or (including conference papers that can be obtained by contacting the author for the original research details) will be included.

A unified data extraction form (an excel spreadsheet) will be produced after discussion within review group. Extracted information will include: characteristics and methodology of included studies, participant characteristics of included studies, details of interventions and control measures, data information for outcome indicators of included studies, assessment of the risk of bias and other information. Two review authors (Liang Dong, Junjun Li) will independently conduct data extraction exercises before the formal data extraction. All differences will be discussed and resolved with the third reviewer (Yulin Li).

The content of data extraction is as follows.

(1) General characteristics: name of first author, published year, tittle, nation or country, contact information.
(2) Information of studies: study design, sample size, randomized information, assignment hiding, blind method, diagnostic criteria, outcome indicators, safety indicators, statistical methods, information of outcome indicators, follow-up time.
(3) Information of participants: age, severity of disease, course of disease, baseline level, complication, healthy condition.
(4) Information of control group: The packaging, shape, taste and color of the oral drug should be consistent with that of the treatment group, and neither the researcher nor the participants could distinguish them.
(5) Outcome indicators: Detailed statistics of sperm quality parameters, including sperm concentration, sperm motility, sperm morphology, sperm DNA fragmentation index, number per ejaculate, sperm viability, data of adverse events and specific information.
(6) Risk of bias: random sequence generation, allocation concealment, blinding of participants and personnel, incomplete outcome data, selective reporting, other bias.^[13]^
(7) Other study information: funding situation, conflict of interest.

If there are abstracts of the conference papers meeting the inclusion criteria, those RCTs will be included in the analysis based on the recommendations of Systematic Reviews section 6.2.2.4 in Coch-rane Handbook.^[14]^ When necessary, the review team will contact the original research author via email to obtain the full text or relevant information.

If there are any questions or puzzles about the original research in the process of data extraction, we will contact the author through email to get specific answers.

### 3.5 Risk of bias assessment

These biases including selection bias, performance bias, detection bias, attrition bias and reporting bias will be assessment based on the Cochrane Collaboration Network Risk Assessment Tool. Two reviewers will independently evaluate and cross check the risk of bias. Discrepancies on the risk of bias will be resolved through discussion with a third review author.

Assessment items include random sequence generation, allocation concealment, blinding of participants and personnel, blinding of outcome assessment, incomplete outcome data, selective reporting and other bias.^[13]^ Each item of bias situation includes low risk, unclear and high risk.^[13]^ Since we cannot determine the authenticity of blinding, the outcome indicators of the systematic review are relatively objective. We define the generation of random sequence, allocation concealment and incomplete data as key domains of risk of bias evaluation. The risk of bias assessment chart of inclusion studies will be produced by using Review Manager 5.3 software.

### 3.6 Data analysis and synthesis

Descriptive analysis will be performed when there be heterogeneity between the studies or when the data cannot be synthesized. Meta-analysis (RevMan 5.3 software) will be used when the studies are homogeneous and the data are similar and synthesizable. Dichotomous variable will be pooled as RR and 95% confidence intervals. Continuous variable will be pooled as MD and 95% confidence intervals. We will use Cochran *Q* statistic and *I*^2^ statistic to test the heterogeneity. *P* < .10 is heterogeneous, and *I*^2^ > 50% is significant heterogeneity. A fixed effect model (Mantel-Haenzel method for RR and Inverse Variance for MD) will be used for *I*^2^ < 50%. A random effects model (D-L method) will be used when the heterogeneity is still significant after sensitivity analysis and subgroup analysis. A *P* < .05 of *z* test will be considered statistically significant. Sensitivity analysis will be used to test the stability of the results. Publication bias will be measured by using a funnel plot (by RevMan 5.3 software) or Egger test (by Stata software).

### 3.7 Subgroup analysis

Subgroup analysis will be performed if the data is sufficient and there is heterogeneity between studies, and the analysis was performed according to different age, ethnicity, kinds of male infertility, interventions, control measures, measurement methods or time of measurement.

### 3.8 Sensitivity analysis

Sensitivity analysis will be used to test the reliability and stability of the meta-analysis result, and to detect the source of heterogeneity. This can be done by excluding trials with a high risk of bias or eliminating each study individually. Then the meta-analysis will be performed again and the results compared with the previous meta-analysis^[]^.

### 3.9 Publication bias

Published bias will be measured by using a funnel plot (by Review Manager 5.3 software), Begg test and Egger test (by Stata software 14.0).^[15,16]^

## 4 Discussion

Due to the great variability of infertility rate, the true incidence of male infertility is not clear.^[17]^ Nearly half of the infertile couples are caused by men.^[1]^

There are a variety of drugs currently used to treat male infertility, including Proxeed, acetyl-L-carnitine, L-carnitine fumarate, Glutathione, Vitamins E and C, Carnitines, Coenzyme-Q10, N-acetylcysteine, Selenium, Zinc, folic acid, and so on, the most important of which is antioxidant.^[3,18]^ Antioxidants have been shown to reduce ROS-induced sperm damage, and can improve sperm quality.^[11,19]^ Due to the solubility of water and fat, ALA is considered as an “universal antioxidant” and one of the most powerful biological antioxidants.^[5]^

The researchers have found that histomorphometric parameters of the seminiferous and epididymal tubules did not show improvement in diabetic rats, which were supplemented with ALA mixed with mash commercial feed, however, there was an improvement in the sperm concentration, sperm motility and percentage of spermatic pathologies.^[4]^ A randomized, triple-blind, placebo-controlled clinical trial showed that the total sperm count, sperm concentration, and sperm motility levels were significantly increased compared with baseline values in ALA intervention group and also higher than the control group. And ALA supplementation also significantly improved semen total antioxidant capacity (TAC) and malondialdehyde levels compared with placebo.^[20]^

Although ALA has excellent antioxidant properties, its clinical application in andrology, especially in spermatogenesis is very limited. Therefore, the purpose of this systematic review is to evaluate the efficacy of ALA as an antioxidant in the treatment of male infertility, and to provide medical evidence for urologists and andrologists. This will be the greatest value in this study.

The systematic review still has some limitations: first, the literature we searched is limited to Chinese and English due to the limitation of language, may cause certain retrieval omissions. Secondly, the sample size of RCT was very low at present, which also leads to the lack of reliability of the conclusion. We look forward to better and larger RCTs to confirm the effect of ALA on sperm.

## Author contributions

**ALA related expertise is provided:** Liang Dong, Xujun Yu, Junjun Li

**Data management:** Liang Dong, Yulin Li, Junjun Li

**Draft writing:** Liang Dong, Xiaojin Zhang, Yulin Li

**Manuscript modification and editing:** Liang Dong, Xiaojin Zhang, Yulin Li

**Methodology:** Liang Dong, Yulin Li, Fang Yang, Junjun Li, Xiaojin Zhang

**Program management:** Liang Dong, Yulin Li

**Research design and concept:** Liang Dong, Xiaojin Zhang, Yulin Li

**Resource:** Xujun Yu, Yulin Li

**Review the manuscript and approve the release:** Liang Dong, Xujun Yu, Yulin Li

**Software:** Yulin Li, Junjun Li, Liang Dong

Liang Dong orcid: 0000-0002-6628-2106.
